# Changes in Disparity in County-Level Diagnosed Diabetes Prevalence and Incidence in the United States, between 2004 and 2012

**DOI:** 10.1371/journal.pone.0159876

**Published:** 2016-08-03

**Authors:** Sundar S. Shrestha, Theodore J. Thompson, Karen A. Kirtland, Edward W. Gregg, Gloria L. Beckles, Elizabeth T. Luman, Lawrence E. Barker, Linda S. Geiss

**Affiliations:** Centers for Disease Control and Prevention, Division of Diabetes Translation, Atlanta, Georgia, United States of America; Texas Tech University Health Science Centers, UNITED STATES

## Abstract

**Background:**

In recent decades, the United States experienced increasing prevalence and incidence of diabetes, accompanied by large disparities in county-level diabetes prevalence and incidence. However, whether these disparities are widening, narrowing, or staying the same has not been studied. We examined changes in disparity among U.S. counties in diagnosed diabetes prevalence and incidence between 2004 and 2012.

**Methods:**

We used 2004 and 2012 county-level diabetes (type 1 and type 2) prevalence and incidence data, along with demographic, socio-economic, and risk factor data from various sources. To determine whether disparities widened or narrowed over the time period, we used a regression-based *β*-convergence approach, accounting for spatial autocorrelation. We calculated diabetes prevalence/incidence percentage point (ppt) changes between 2004 and 2012 and modeled these changes as a function of baseline diabetes prevalence/incidence in 2004. Covariates included county-level demographic and, socio-economic data, and known type 2 diabetes risk factors (obesity and leisure-time physical inactivity).

**Results:**

For each county-level ppt increase in diabetes prevalence in 2004 there was an annual average increase of 0.02 ppt (p<0.001) in diabetes prevalence between 2004 and 2012, indicating a widening of disparities. However, after accounting for covariates, diabetes prevalence decreased by an annual average of 0.04 ppt (p<0.001). In contrast, changes in diabetes incidence decreased by an average of 0.04 ppt (unadjusted) and 0.09 ppt (adjusted) for each ppt increase in diabetes incidence in 2004, indicating a narrowing of county-level disparities.

**Conclusions:**

County-level disparities in diagnosed diabetes prevalence in the United States widened between 2004 and 2012, while disparities in incidence narrowed. Accounting for demographic and, socio-economic characteristics and risk factors for type 2 diabetes narrowed the disparities, suggesting that these factors are strongly associated with changes in disparities. Public health interventions that target modifiable risk factors, such as obesity and physical inactivity, in high burden counties might further reduce disparities in incidence and, over time, in prevalence.

## Introduction

The health and economic burdens of diabetes have been well documented and are major public health concerns [1, 2]. In recent decades, both prevalence and incidence of diabetes have increased in the United States. From 1980 to 2012, the prevalence of age-adjusted diagnosed diabetes increased from 3.7% to 8.4% among the U.S. civilian population aged ≥18 years [3]. Over the same period, among adults aged 18–79 years the annual age-adjusted diabetes incidence increased from 3.5 per 1000 per year to 7.1 per 1000 [4]. It has been projected that diabetes prevalence would increase to as high as 33% and diabetes incidence would increase to 15 per 1000 of the U.S. adults population by 2050 [5]. While diabetes burden is still high, a recent analysis showed that diabetes prevalence and incidence leveled off during 2008–2012 [6].

In addition to the high burden, geographical disparities exist between counties for both prevalence and incidence of diabetes [7–13]. A ‘diabetes belt’, or a swath of contiguous counties with high diabetes prevalence, primarily located in the South, was previously described [8]. In addition, spatial clusters of low prevalence were found in the Midwest [13]. However, the extent to which disparities in diabetes prevalence and incidence across U.S. counties have changed over time is not known.

Elimination of health disparities, including those based on geographic location, is an overarching goal of the U.S. Healthy People 2020 initiative to help all population groups achieve high levels of health [14]. Examining the changes in geographic variation in diabetes outcomes is essential for monitoring progress in achieving disparity goals, as well as assessing the impact of past policy efforts and guiding decisions to prioritize future interventions.

The focus of this study was to determine whether county-level disparities in diabetes prevalence and incidence (type1 and type 2) have been widening or narrowing over time. We used a regression-based *β*-convergence approach to examine changes in disparities in the U.S. county-level diabetes prevalence and incidence between 2004 and 2012 relative to their starting levels. Because national level findings may not apply to lower geographic levels, we also conducted similar analyses by the U.S. census region and by state.

## Methods

This study is solely based on the publicly available estimated county-level data from the United States. It did not involve human participant, specimens or tissue samples, or vertebrate animals, embryos or tissues. Therefore, this study did not require an approval from institutional review board.

### Counties Assessed

We analyzed data from all 3109 counties in the 48 contiguous states and the District of Columbia. We excluded Alaska and Hawaii because the outcome variables are spatially auto-correlated, preventing calculation of significance levels in non-contiguous areas. (http://geodacenter.asu.edu/node/402#lisaisle, Accessed August 2, 2015).

### Variables

County-level estimates of diagnosed diabetes (type 1 and type 2 combined) prevalence and incidence for 2004 and 2012 were obtained from data annually published by the Centers for Disease Control and Prevention (CDC) [15, 16]. The starting year (2004) was the first year for which county level data are available. The CDC’s county level estimates of diabetes prevalence and incidence are modeled based on a Bayesian small area estimation approach that uses data from CDC’s Behavioral Risk Factor Surveillance System (BRFSS) and the U.S. Census Bureau’s Population Estimates Program [17, 18].

In 2011, BRFSS began using cell phones in addition to landline phones to interview respondents [19]. Although the inclusion of cell phones appears to have had minimal effect on diabetes prevalence estimates [19], we conducted sensitivity analyses to assess its impact on our analyses.

Covariates included county-level demographic and socio-economic characteristics, and obesity and leisure-time physical inactivity prevalence using data from 2004 if available. If 2004 data were not available we used the data from closest year; 2003 data were used for metropolitan statistical area (MSA) designation of counties and 2000 data were used for educational attainment, because 2004 data were not available. Demographic factors included age distribution (percentage of adults 20–43 years (reference), 44–64 years, or ≥65 years) and sex (percentage of the population aged ≥20 years that were female). These variables were from the U.S. Census Bureau population estimates data [20]. Socioeconomic factors included median household income from the U.S. Census Bureau Small Area Income and Poverty Estimates [21] (in natural logarithm because of skewedness); MSA designation of counties, from the United States Department of Agriculture County typology codes [22]; and educational attainment (percentage of adults age 25 and older with less than high school degree (reference), high school, some college or associate degree, or bachelor’s degree or higher) and race/ethnicity (percentage of adults non-Hispanic white (reference), non-Hispanic black or African American (hereafter we use the term non-Hispanic black), Hispanic, non-Hispanic Asian, and “other race/ethnicity”) from the U.S. Census Bureau. Diabetes risk factors included the percentage of adults who were obese (defined as having a body mass index of ≥30 kg/m^2^ based on self-reported height and weight) [23] and the percentage of adults who self-reported engaging in no leisure-time physical activity [24]. Both of these are estimated from CDC.

### Analytic Approach

To explore geographical patterns of change, we mapped the percentage-point (ppt) differences between 2004 and 2012 in diabetes prevalence and incidence estimates in adults for each county. To determine whether disparities widened or narrowed over the time period, we modeled these changes as a function of the baseline (2004) prevalence and incidence. Specifically, we used regression-based β-convergence, an approach commonly used to analyze “economic convergence”, in which areas that have low initial levels of economic development achieve higher growth rates over time than those with initially high levels of economic development [25]. Recently, this concept has also been applied to measuring changes in disparity between populations in health care expenditures [26–29] and health outcomes [30]. With this method, we examined the *β* coefficient derived from a regression model that measures changes in diabetes prevalence and incidence between 2004 and 2012, relative to the diabetes prevalence and incidence at the start of the time interval (2004). In models that do not control for other covariates, the *β* coefficient reflects absolute widening or narrowing of county-level disparities; in models that control for covariates, the *β* coefficient reflects conditional widening or narrowing of county-level disparities [26, 30].

We carried out a series of tests to determine the appropriate regression models. First, we determined whether an ordinary least squares regression was appropriate by testing the spatial autocorrelation (i.e., if the rate changes in a county are correlated with neighboring counties) in diabetes prevalence and incidence using the global Moran’s I statistics [31, 32]. We defined immediately contiguous neighbors using the first-order Queen Contiguity spatial weight matrix and standardized the weights so that rows summed to 1.0 [31]. The analysis showed that changes in diabetes prevalence and incidence between 2004 and 2012 were spatially auto-correlated, suggesting the need to use a spatial regression model rather than an ordinary least squares regression model, because estimates from the latter would suffer from biases and inconsistencies [33]. Next, to determine an appropriate spatial regression model, we used model selection criteria suggested by Anselin [34]. We determined that a spatial lag model would be appropriate for determining changes in disparity in diabetes prevalence. This model accounts for spatial autocorrelation in the outcome between one county and its neighboring counties. However, a spatial error model was better fit for determining changes in disparity in diabetes incidence. This model accounts for spatial autocorrelation in the residuals between a county and its neighboring counties, which can result from omitted variables related to the outcome variable.

For changes in diabetes prevalence and incidence, we first examined unadjusted models (Model I) and noted whether disparities were widening or narrowing. We then examined a series of adjusted models, first including demographic factors (Model II), then adding socio-economic and behavioral risk factors (Model III), to understand the relative contribution of each factor in explaining the change of diabetes prevalence and incidence.

We examined the following unadjusted (Eq 1) and adjusted (Eq 2) spatial lag models for changes in diabetes prevalence:

$${DM}_{i,T-0}=\alpha +{\psi W*DM}_{i,T-0}+{\beta DM}_{i,0}+\varepsilon_{i}$$ (1)

$${DM}_{i,T-0}=\alpha +{\psi W*DM}_{i,T-0}+{\beta DM}_{i,0}+{\phi X}_{i,0}+\varepsilon_{i}$$ (2)

where *DM*_*i*,*T–0*_ is the ppt change in diabetes prevalence of *i*^*th*^ county between 2004 and 2012. W*DM_*i*_,_*T–0*_ is the spatially weighted *DM*_*i*,*T–0*,_ adjusting for the *DM*_*i*,*T–0*_ of immediate neighbors, where *W* is the first order Queen Contiguity Weight matrix used to identify immediate neighbors [31]. *ψ* is the coefficient for spatial lag term. *DM*_*i*,*0*_ is diabetes prevalence in the *i*^*th*^ county in 2004 (i.e., the independent variable). *ε*_*i*_ is the uncorrelated error term for the *i*^*th*^ county. *X*_*i*,*0*_ represents county-level covariate factors in the initial year and ϕ represents the effect of these factors on the outcome. *β* is the convergence coefficient; disparity is narrowing if *β*<0, widening if *β*>0, and not changing if *β* = 0.

We examined the following unadjusted (Eq 3) and several adjusted (Eq 4) spatial error models, for changes in diabetes incidence:

$${DM}_{i,T-0}=\alpha +{\beta DM}_{i,0}+\varepsilon_{i},\;where\;\varepsilon =\lambda W\varepsilon +\xi$$ (3)

$${DM}_{i,T-0}=\alpha +{\beta DM}_{i,0}+{\phi X}_{i,0}+\varepsilon_{i},\;where\;\varepsilon =\lambda W\varepsilon +\xi$$ (4)

These specifications differ from those used for the diabetes prevalence models in the lag and error terms. The error term, *ε*, is now the correlated spatial lagged error term, *Wε* is spatially weighted vector of error terms, *ξ* the vector of uncorrelated error terms, and *λ*, the coefficient for spatial error term.

For analyses by census region, we tested both unconditional (Model I) and conditional *β*-convergence including all the covariates, accounting for spatial autocorrelation as we did for national models (Model III). We used an ordinary least squares regression if spatial autocorrelation in the outcomes was not observed. For analyses by state, we tested only unconditional *β*-convergence without accounting for spatial autocorrelation, because some states have too few counties to support the full analysis.

After estimating *β* coefficients using the models specified above, we calculated average annual ppt changes in diabetes prevalence and incidence by dividing the coefficients by 8 (the number of years between 2004 and 2012).

### Sensitivity analysis

In order to examine if the inclusion of cellphones by BRFSS beginning in 2011 had an impact on our results, we examined trends in the medians of county estimates of age-adjusted diabetes prevalence and incidence. We also examined how our results might change by using 2010 data rather than 2012 data in our models (i.e., using 2004 and 2010 data).

We used Stata v.13 (StataCorp LP; College Station, Texas) for summary statistics and GeoDa 0.95 software [31] to test for the spatial autocorrelation in outcome variables and regression analyses.

## Results

### Descriptive Statistics

Average county-level characteristics of counties studied are presented in Table 1. Average county-level diabetes prevalence increased 2.9 ppt (95% CI: 2.85–2.94) from 8.3% in 2004 to 11.2% in 2012 (average annual increase 0.36 ppt) (Table 1). Average county-level diabetes incidence decreased 0.08 ppt (95% CI: 0.08–0.09), from 1.03% to 0.94% (average annual decrease 0.01ppt).

**Table 1:** Average county-level characteristics.

Variables	Mean	SD	Minimum	Maximum
Diabetes prevalence (%), 2004	8.3	1.6	3.0	14.6
Diabetes prevalence (%), 2012	11.2	2.4	3.6	23.5
Diabetes incidence (%), 2004	1.0	0.2	0.4	2.1
Diabetes incidence (%), 2012	0.9	0.2	0.3	2.4
Demographic factors:				
Aged 20–43 years (%), 2004	44.2	7.1	22.2	86.8
Aged 44–64 years (%), 2004	35.3	3.4	10.3	54.0
Aged ≥65 years (%), 2004	20.5	5.2	2.9	44.8
Female (%), 2004	51.0	2.6	26.2	60.2
NH white (%), 2004	82.6	17.7	2.7	99.6
NH black (%), 2004	8.3	13.5	0.0	83.5
Hispanic (%), 2004	6.0	11.4	0.1	96.8
NH Asian (%), 2004	0.9	1.8	0.0	30.7
NH American Indian (%), 2004	1.4	5.6	0.0	85.7
Other races (%), 2004	0.8	0.7	0.0	10.0
Socio-economic factors:				
Median household income ($), 2004	38,045	9,566	17,787	94,658
Less than high school degree (%), 2000	22.7	8.7	3.0	65.3
High school, some college or associate degree (%), 2000	60.8	7.0	27.6	81.1
Bachelor degree or higher (%), 2000	16.5	7.8	4.9	63.7
Metro counties ^**a**^, 2003	0.4	0.5	0.0	1.0
Risk factors:				
Obesity prevalence (%), 2004	26.2	3.4	11.7	38.9
Physical inactivity (%), 2004	26.0	5.2	9.2	42.4

NH non-Hispanic; SD standard deviation (from mean)

^**a**^ counties are in a metropolitan statistical area.

Largest average increases in diabetes prevalence were in the South census region (3.3 ppt) and smallest in the West (2.2 ppt). Largest average decreases in diabetes incidence were in the Midwest (0.09 ppt) and smallest in the Northeast (0.04 ppt) (results not shown).

**Fig 1:**
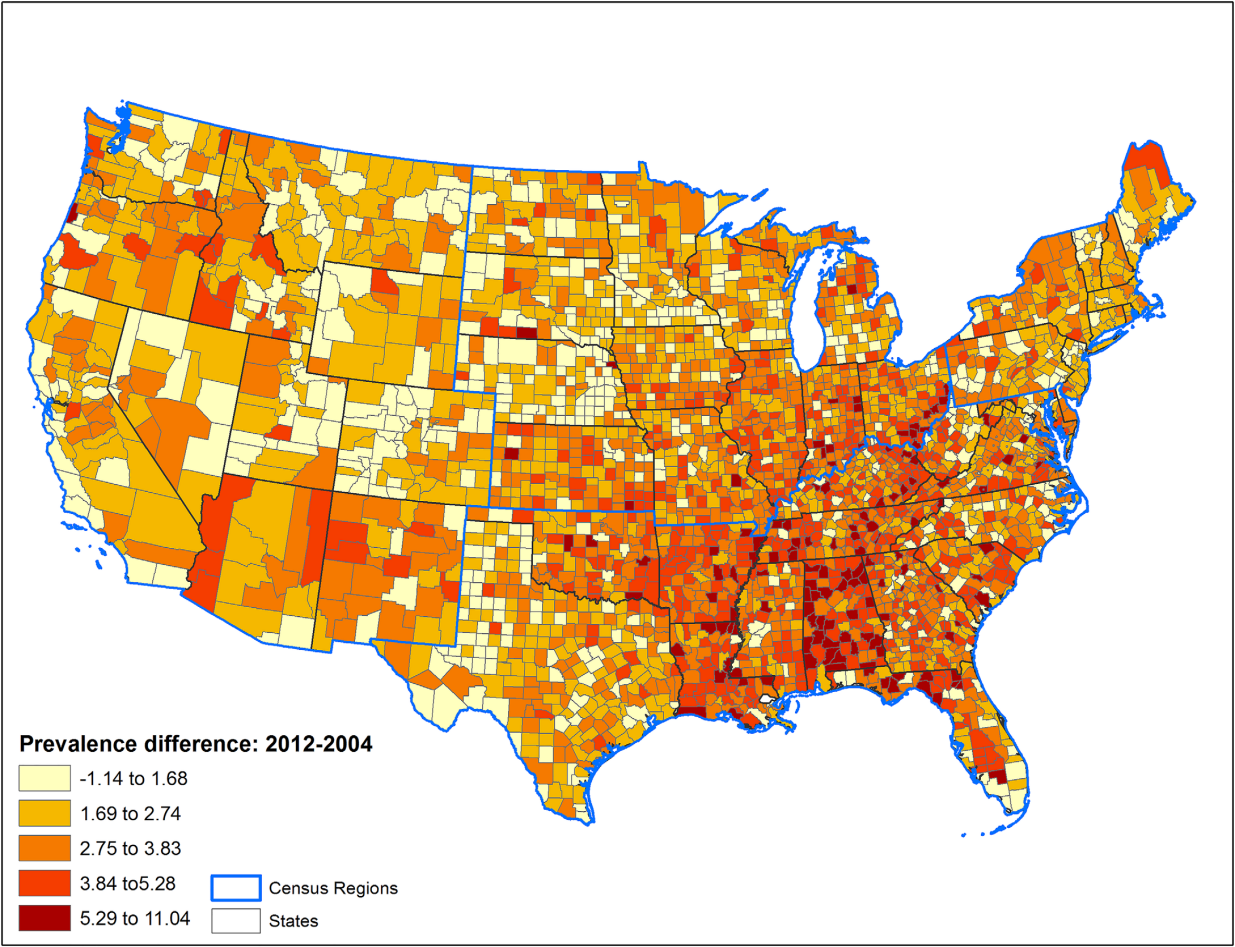
Percentage point change in county-level prevalance of diagnosed diabetes among U.S. adults between 2004 and 2012.

**Fig 2:**
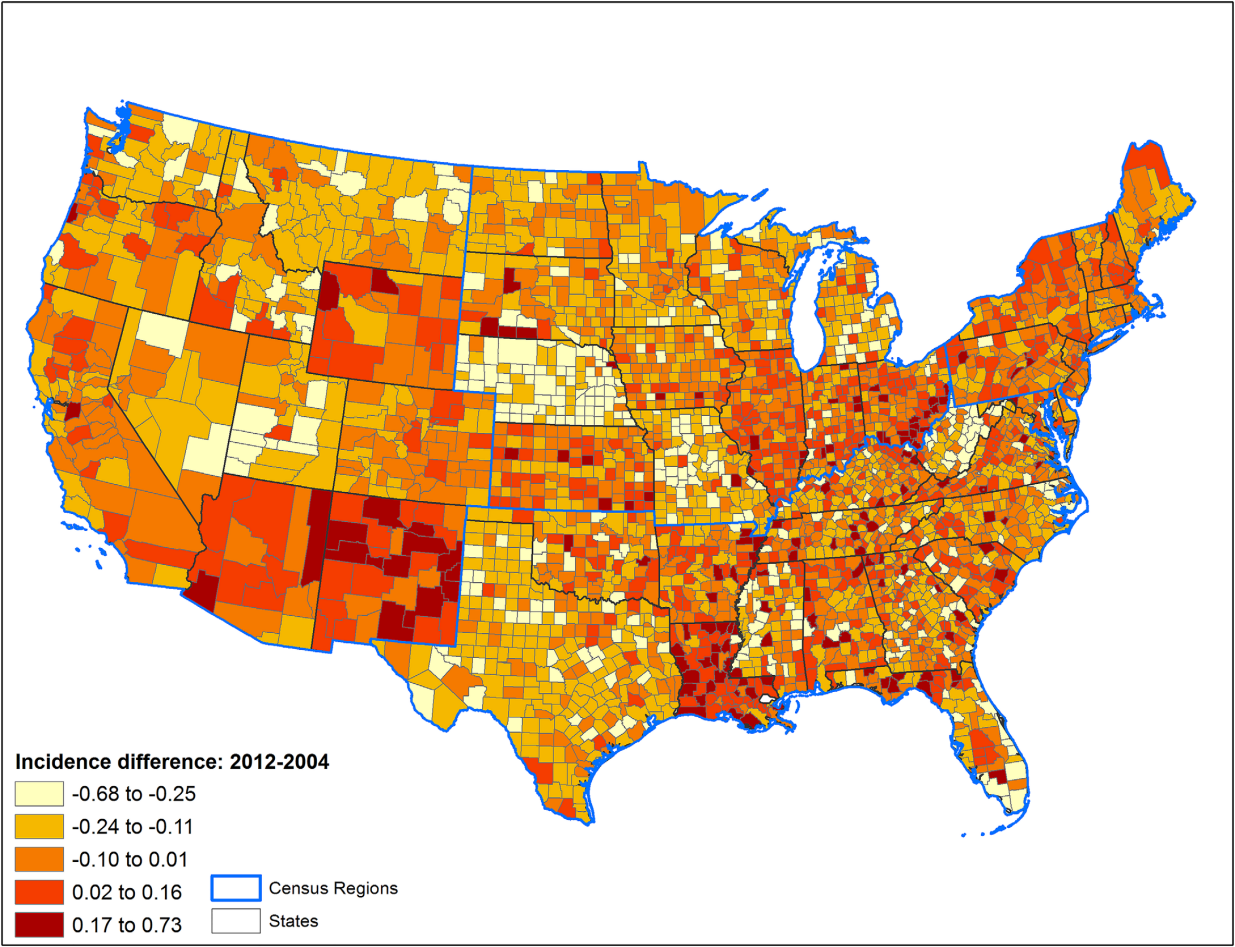
Percentage point changes in county-level incidence of diagnosed diabetes among U.S. adults between 2004 and 2012.

The patterns of county-level changes in diabetes prevalence and incidence between 2004 and 2012 are presented in Figs 1 and 2, respectively. Moran’s I statistic for change in diabetes prevalence between 2004 and 2012 was 0.32 (p<0.001), and for incidence was 0.26 (p<0.001), indicating existence of significant spatial autocorrelation in these outcomes between counties.

### Changes in Disparity in Diabetes Prevalence: National Average

The unadjusted *β* coefficient for change in diabetes prevalence between 2004 and 2012 was positive: 0.15 ppt (annually 0.02 ppt; p<0.001) for each ppt increase in prevalence in 2004. Thus, county level disparity in diabetes prevalence widened over this time period (Table 2, Model I). After controlling for demographic factors, the coefficient was lower but still positive: 0.08 (annually 0.01 ppt; p<0.001), indicating that demographic factors partly explained the widening disparity in diabetes prevalence (Table 2, Model II). When socio-economic and behavioral covariates were also added, the coefficient was negative: -0.32 (annually -0.04 ppt; p<0.001) (Table 2, Model III), indicating that disparities narrowed after accounting for these factors.

**Table 2:** Estimates of percentage point change in county-level diabetes prevalence between 2004 and 2012.^†^

Variables	Model I		Model II		Model III	
	*β* Coef.	Z-Val	*β* Coef.	Z-Val	*β* Coef.	Z-Val
Constant	0.32**	20.2	0.93*	2.0	-1.36	-0.9
Diabetes prevalence (%), 2004	0.15***	2.9	0.08***	4.0	-0.32***	-12.1
Changes in diabetes prevalence of neighboring counties (%), 2004–2012	0.45***	11.2	0.43***	19.3	0.33***	14.4
Aged 44–64 years (%), 2004			0.02**	3.2	0.05***	7.5
Aged ≥65 years (%), 2004			-0.01	-1.9	0.01	1.9
Female (%), 2004			-0.01	-1.1	0.01	1.6
NH black (%), 2004			0.01***	4.5	0.02***	7.7
Hispanic (%), 2004			-0.01***	-3.3	-0.01***	-5.8
NH Asian (%), 2004			-0.09***	-7.0	0.01	0.9
NH American Indian (%), 2004			0.01	1.8	0.02***	5.3
Other races (%), 2004			0.06	1.8	0.07*	2.2
Metro counties (= 1), 2003			-0.05	-1.1	0.15**	3.0
High school, some college or associate degree (%), 2000					-0.02***	-5.3
Bachelor or higher, (%), 2000					-0.07***	-13.7
*Log* median household income($), 2004					0.25	1.6
Obesity prevalence (%), 2004					0.05***	4.3
Physical inactivity (%), 2004					0.07***	10.5
R^2^	0.24		0.27		0.38	
Annual average percentage point change for each percent of DM prevalence in 2004	0.02***		0.01***		-0.04***	

^**†**^ Based on spatial lag regression models.

Model I: Not controlling for covariates; Model II: controlling for distribution of demographic factors; Model III: controlling for distribution of demographic, socio-economic, and diabetes risk factors (obesity and leisure-time physical inactivity).

NH non-Hispanic; R^2^ coefficient of determination.

p-values

* <0.05

** <0.01

*** <0.001

Diabetes prevalence increased more in counties with a higher percentage of non-high school graduates, non-Hispanic blacks and non-Hispanic American Indians, and middle-aged adults; those located in MSAs; and in those with a high prevalence of obesity and adults reporting no leisure time physical activity. There was significant spatial autocorrelation in the change in diabetes prevalence between neighboring counties (Table 2, Model III).

### Changes in Disparity in Diabetes Incidence: National Average

The unadjusted *β* coefficient for change in diabetes incidence between 2004 and 2012 was –0.31ppt (annually -0.04 ppt; p<0.001) for each ppt increase in incidence in 2004, indicating that disparities between counties narrowed (Table 3, Model I). After controlling for demographic factors, the decrease in disparities increased to –0.49 ppt (annually -0.06 ppt; p<0.001) (Table 3, Model II), indicating that these factors contributed to the narrowing disparities. Disparities in diabetes incidence were further decreased to –0.73 ppt when also controlling for socio-economic and behavioral factors (annually -0.09 ppt; p<0.001).

**Table 3:** Estimates of percentage point change in county-level diabetes incidence between 2004 and 2012.^†^

Variables	Model I		Model II		Model III	
	*β* Coef.	Z-Val	*β* Coef.	Z-Val	*β* Coef.	Z-Val
Constant	0.23***	13.8	0.290***	5.9	0.07	0.3
Diabetes incidence (%), 2004	-0.31***	-20.0	-0.490***	-24.7	-0.73***	-34.2
Aged 44–64 years (%), 2004			0.002**	2.8	0.003***	4.3
Aged ≥65 years (%), 2004			-0.001	-0.7	-0.001*	-0.2
Female (%), 2004			0.001	0.8	0.003***	3.7
NH black (%), 2004			0.003***	10.5	0.003***	9.1
Hispanic (%), 2004			-0.001	-1.4	-0.002***	-0.5
NH Asian (%), 2004			-0.014***	-7.4	0.002	0.8
NH American Indian (%), 2004			0.006***	11.2	0.006***	12.3
Other races (%), 2004			-0.002	-0.4	-0.001	-0.3
Metro counties (= 1), 2003			-0.010	-1.5	0.007	1.1
High school, some college or associate degree (%), 2000					-0.003***	-4.1
Bachelor degree or higher (%), 2000					-0.01***	-10.7
*Log* median household income ($), 2004					0.02	0.7
Obesity prevalence (%), 2004					0.01***	5.2
Physical inactivity (%), 2004					0.01***	10.4
Spatial error term (λ)	0.57***	28.5	0.60***	31.2	0.55 ***	27.0
R^2^	0.27		0.33		0.44	
Annual average percentage point change for each percent of DM incidence in 2004	-0.04***		-0.06		-0.09***	

^†^Based on spatial error regression models

Model I: Not controlling for covariates; Model II: controlling for distribution of demographic factors; Model III: controlling for distribution of demographic, socio-economic, and behavioral risk factors.

NH non-Hispanic; R^2^ coefficient of determination.

p-values

* <0.05

** <0.01

*** <0.001

Diabetes incidence decreased more in counties with a higher percentage of high school graduates and a lower percentage of Hispanics, those with a lower percentage of middle-aged adults, and those with a lower percentage of physically inactive and obese adults (Table 3). The significant positive spatial error term suggests that unobserved factors influencing the changes in diabetes incidence in one county were positively associated with the diabetes incidence of neighboring counties.

### Changes in Disparity in Diabetes Prevalence: Region and State Averages

Positive unadjusted *β* coefficients indicated that there were widening disparities in county-level diabetes prevalence in all U.S. census regions (although the change in the Northeast was not statistically significant). Estimated annual rates of increase in diabetes prevalence ranged from 0.01 ppt in the South region to 0.02 ppt in the Midwest and West for each ppt increase in diabetes prevalence in 2004 (Table 4 Model I). When controlling for all covariates the *β* coefficients for each region were negative, indicating a conditional narrowing of disparity in diabetes prevalence as found in national model (Table 4 Model III).

**Table 4:** Estimated annual percentage point changes in county-level diabetes prevalence and incidence between 2004 and 2012 for each percentage point increase in diabetes prevalence or incidence in 2004 by census region.

Census region	No. of counties	Prevalence^1^		Incidence^2^	
		Model I	Model III	Model I	Model III
Northeast ^**3**^	217	0.010	-0.085***	-0.024***	-0.085***
Midwest	1055	0.019***	-0.025***	-0.025***	-0.091***
South	1423	0.014***	-0.076***	-0.045***	-0.096***
West	414	0.019**	-0.025***	-0.036***	-0.091***

*** = p<0.001

** = p<0.01

^1^ The estimates are based on spatial lag models.

^2^ The estimates are based on spatial error models.

^3^ The estimates are from ordinary least squares regression models.

Model I: Not controlling for covariates; Model III: controlling for distribution of demographic, socio-economic, and behavioral risk factors.

A significant positive result indicates widening county-level disparities in the region; a significant negative result indicates narrowing county-level disparities in the region.

**Fig 3:**
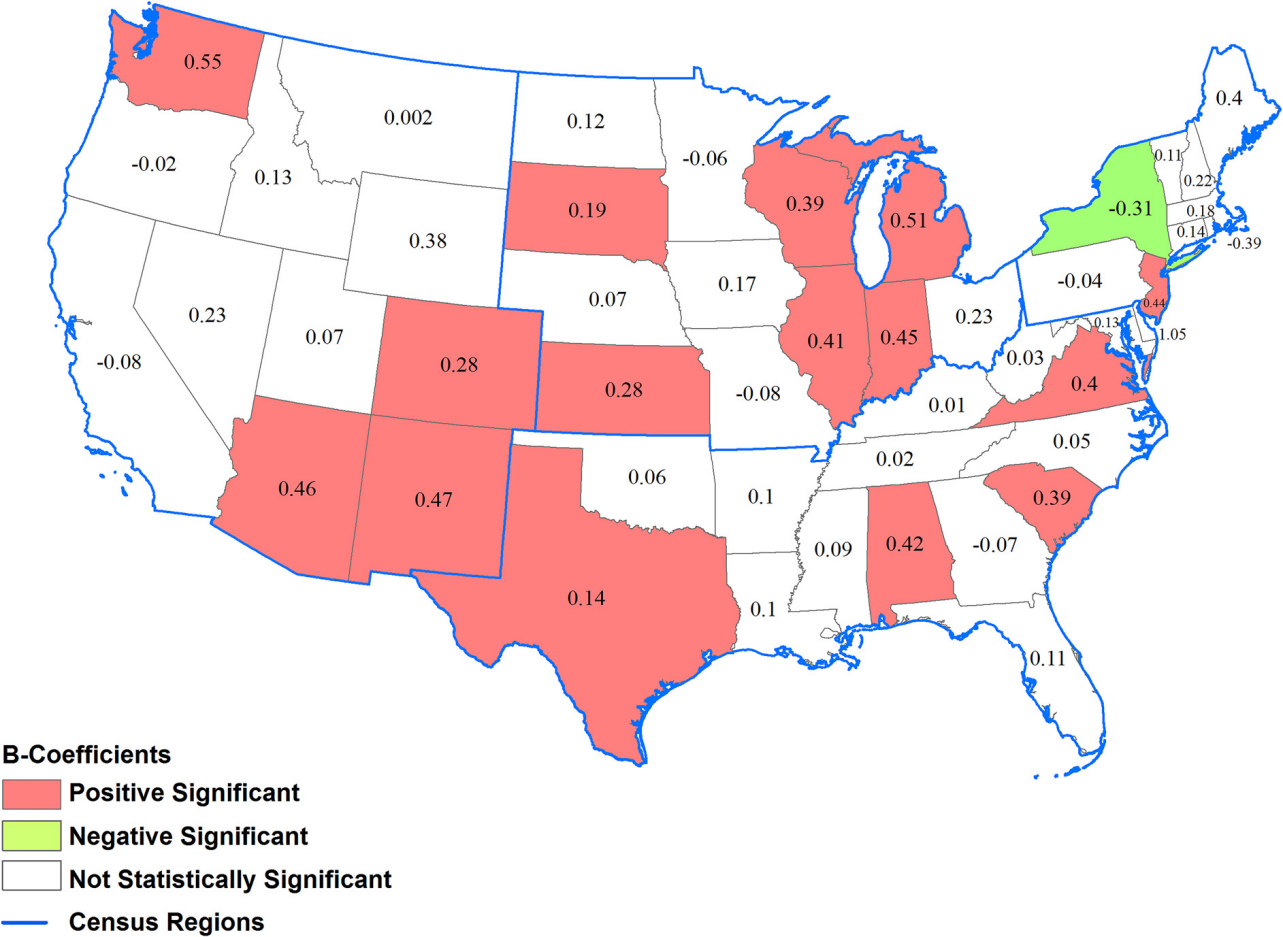
*β*-Coefficients for changes in county-level prevalence of diagnosed diabetes among adults between 2004 and 2012 for a percent point increase in prevalence in 2004 by state. *Positively significant β*-*Coefficient*: county-level disparity in diabetes prevalence within the state widened between 2004 and 2012 (p<0.05); *Negatively significant β*-*Coefficient*: county-level disparity in diabetes prevalence within the state narrowed between 2004 and 2012; *Not statistically significant β*-*Coefficient* (p<0.05); county-level disparity in diabetes prevalence within the state did not change between 2004 and 2012 (p>0.05).

Results within regions varied by state (Fig 3). In the Northeast, only 1 of 9 states (New Jersey) had significant widening disparity in county-level diabetes prevalence. In the Midwest, 6 of 12 states (Illinois, Indiana, Kansas, Michigan, South Dakota, and Wisconsin) had widening disparity. In the South, 4 of 16 states (Alabama, South Carolina, Texas, and Virginia) had widening disparity. In the West, 4 of 11 states (Arizona, Colorado, New Mexico, and Washington) had widening disparity (excluding Alaska and Hawaii, which were not evaluated). New York was the only state that had significant narrowing of county-level disparity in diabetes prevalence. In the remaining 33 states, changes in disparities were not statistically significant (Fig 3).

### Changes in Disparity of Diabetes Incidence: Region and State Averages

We found a narrowing of disparities in county-level diabetes incidence in all regions. Estimated annual rates of decrease in diabetes incidence was 0.02 ppt in the Northeast, 0.03 ppt in the Midwest, 0.04 ppt in the West and 0.05 ppt in the South for each ppt increase in diabetes incidence in 2004 (Table 4).

**Fig 4:**
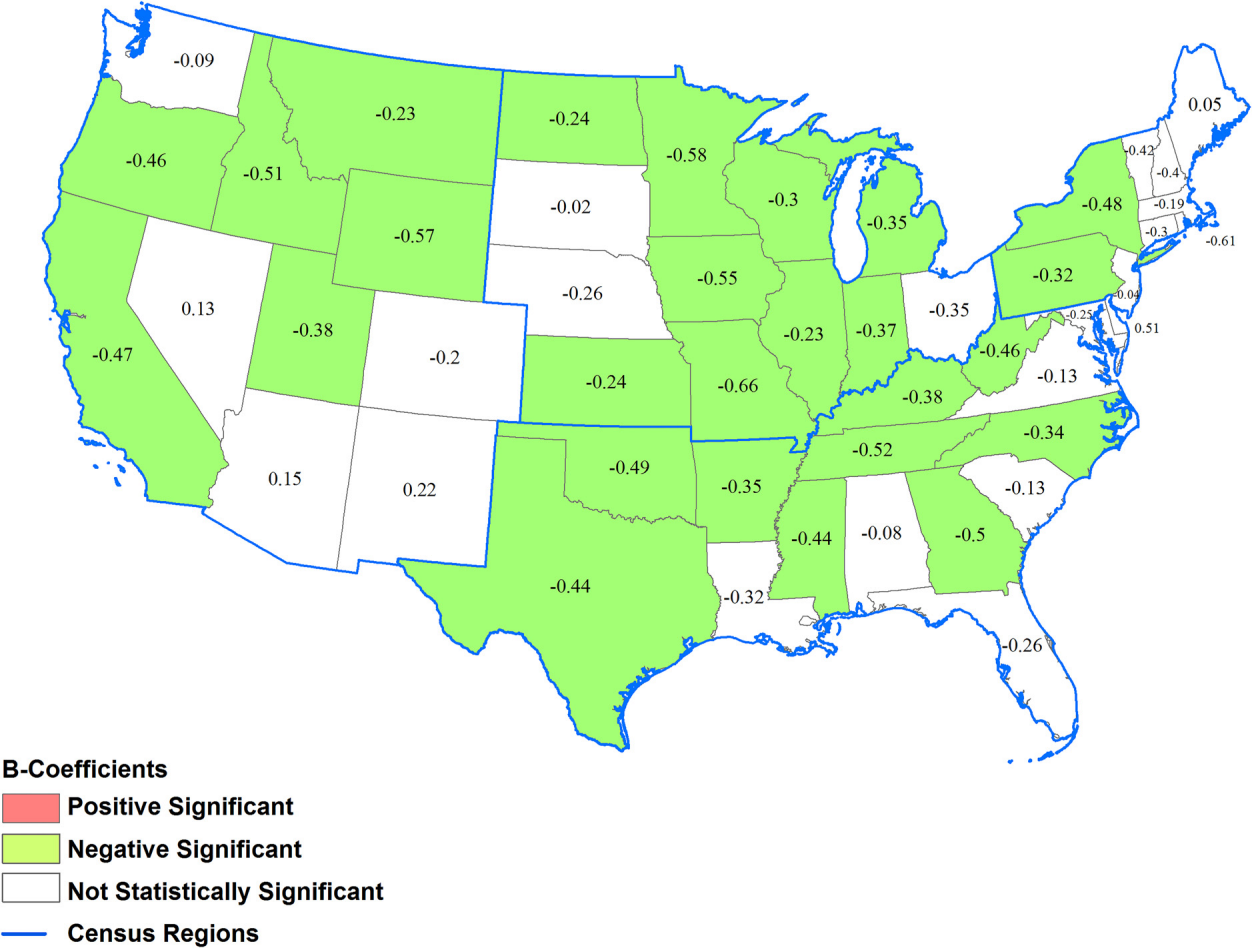
*β*-Coefficients for changes in county-level incidence of diagnosed diabetes among adults between 2004 and 2012 for a percent point increase in incidence in 2004 by state. *Positively significant β*-*Coefficient*: county-level disparity in diabetes incidence within the state widened between 2004 and 2012 (p<0.05); *Negatively significant β*-*Coefficient*: county-level disparity in diabetes incidence within the state narrowed between 2004 and 2012; *Not statistically significant β*-*Coefficient* (p<0.05); county-level disparity in diabetes incidence within the state did not change between 2004 and 2012 (p>0.05).

State-level average changes varied widely within regions. In the Northeast, New York and Pennsylvania had significantly narrowing disparity in diabetes incidence. In the Midwest, disparities narrowed in all states except Nebraska, Ohio, and South Dakota. In the South, a narrowing of disparities was observed in Arkansas, Georgia, Kentucky, Mississippi, North Carolina, Oklahoma, Tennessee, Texas, and West Virginia. In the West, disparity narrowed in California, Idaho, Montana, Oregon, Utah, and Wyoming. In the remaining 22 states, changes in disparities were not statistically significant (Fig 4).

### Sensitivity Analysis

An examination of trends in the medians of age-adjusted county-level diabetes prevalence and incidence, revealed no change in trend in 2011, the year cell phones were introduced. Also, when we estimated the changes between 2004 and 2010 data, for each ppt increase in prevalence in 2004 the annual percentage point change for the unadjusted model (Model I) of diabetes prevalence was 0.02 (p<0.001) and for diabetes incidence was -0.04 (p<0.001), identical to the results using 2004 and 2012 data. Results for the adjusted models (Model III) were also nearly identical, the annual percentage point change for prevalence was -0.05 (p<0.001) (2010 data) vs. -0.04 (2012 data) and for incidence it was -0.11 (p<0.001) (2010 data) vs. -0.09 (2012 data).

## Discussion

We found that county-level disparities in diagnosed diabetes prevalence in the United States widened between 2004 and 2012, while disparities in incidence narrowed. However, results varied by census region and state. Although prior studies have noted geographic disparities in county-level diabetes prevalence and incidence [8, 13], to our knowledge this is the first to examine whether these disparities have changed over time. Analyzing rates of change relative to the initial condition allows us to determine if counties with high prevalence and incidence are worsening, or if they are becoming more similar to the rest of the counties.

Our findings may be explained by several key factors. The decreased county-level disparity in incidence may be due to a greater reduction in diabetes risk factors in counties with higher initial burden. A recent national-level study found a leveling off of diabetes incidence during 2008–2012 [6]. Targeted diabetes prevention programs in the U.S. including lifestyle interventions for high risk populations, have shown promising results in reducing diabetes risk and may have played a role in reducing the level and disparities [35–37]. Furthermore, several studies have demonstrated a narrowing of the gap in state-level health care expenditures, especially in physician and other professional services and home health care [26]. If these trends continue, they may lead to an eventual narrowing of the disparity in prevalence.

Prevalence measures are composed of a mixture of both long-term and recent cases, and the interplay of diabetes incidence and mortality on diabetes prevalence has been previously discussed [38]. The increased disparity in prevalence could have been driven by a decline in the rate and disparities in diabetes-related mortality across the counties. In recent decades, the United States has witnessed substantial decline in death rates among people with diabetes [39]. Although diabetes incidence has declined, diabetes prevalence will continue to rise if reductions in mortality outweigh reductions in new cases [38]. Furthermore, geographic disparities will widen if disparities in diabetes mortality are reduced at a greater rate than disparities in incidence.

Accounting for demographic characteristics attenuated the widening disparities observed in prevalence, and increased the narrowing of disparities in incidence. Moreover, when socio-economic and behavioral risk factors were additionally considered, the disparities further narrowed, suggesting that these factors are strongly associated with changes in disparities. Educational attainment, prevalence of obesity, and leisure time physical inactivity are modifiable factors contributing to disparities in both prevalence and incidence. Several studies have shown that socio-economic status and educational attainment are significant predictors of diabetes incidence [40, 41]. One Danish longitudinal study suggested that the incidence of diabetes could be lowered by reducing socio-economic inequality [42]. Obesity and physical inactivity are also well-known risk factors for diabetes [43], and have been shown to mediate the effect of education [44]. Thus public health interventions targeting counties that have populations with low educational attainment and high rates of obesity and physical inactivity might decrease diabetes burden and county-level disparities. Furthermore, our finding of significant spatial autocorrelation suggests that the effects of public health interventions may occur not only in the target counties but also spill over to surrounding counties.

### Strengths and Limitations

This is the first study to examine changes in county-level disparities in diabetes prevalence and incidence in the United States. A key methodological strength lies in the augmentation *β*-convergence models to account for significant spatial autocorrelation in the changes in diabetes prevalence/incidence among U.S. counties. Previous studies using *β*-convergence models to examine changes in disparity of health outcomes were primarily based on ordinary least square regression models and did not test for spatial autocorrelation in the outcomes across the geographical units [30]. This point is critical because, in the presence of spatial autocorrelation, estimates from ordinary least square regression models are biased and inconsistent [33].

Our study had several limitations. First, the data used did not allow us to account for undiagnosed diabetes or distinguish between type 1 and type 2 diabetes. Factors such as obesity and leisure-time physical inactivity are only associated with type 2 diabetes; however, as approximately 95% of all diabetes cases among adults are type 2, the conclusions apply to the great majority of cases [45]. Second, our analyses are subject to the limitations of ecological analyses, and county-level results may not be applicable to individuals. Third, our study covered a limited time period. Because prevalence includes both long-term and recent cases, analysis using more years of data would likely provide better insight into changes in diabetes prevalence. Fourth, demographic, socio-economic, and behavioral factors, as well as diabetes status, were self-reported and are subject to non-response, recall and social desirability bias [8], which might have varied geographically. In addition, we did not account for changes in these factors over time, which may not have been uniform over all counties. Fifth, the introduction of cell phones use by BRFSS in 2011 could have impacted our analyses; however, our sensitivity analyses suggest that this survey change had little effect on our results. Finally, we used data from 2004 and 2012 only, and our fitted model results reflected average annual changes; actual changes might not have been linear.

## Conclusions

Between 2004 and 2012, disparities in U.S. county-level diabetes prevalence increased. However, baseline demographic, socio-economic, and behavioral characteristics of county populations accounted for the increased disparity. Over the same time period, cross-county disparities in diabetes incidence declined. Public health interventions targeting counties with high prevalence of obesity and physical inactivity might further reduce county-level disparities in incidence and, over time, in prevalence.
